# Repurposing p97 inhibitors for chemical modulation of the bacterial ClpB–DnaK bichaperone system

**DOI:** 10.1074/jbc.RA120.015413

**Published:** 2020-11-21

**Authors:** Przemyslaw Glaza, Chathurange B. Ranaweera, Sunitha Shiva, Anuradha Roy, Brian V. Geisbrecht, Frank J. Schoenen, Michal Zolkiewski

**Affiliations:** 1 Department of Biochemistry and Molecular Biophysics, Kansas State University, Manhattan, Kansas, USA; 2 High Throughput Screening Laboratory, University of Kansas, Lawrence, Kansas, USA; 3 Lead Development and Optimization Shared Resource, University of Kansas Cancer Center, Kansas City, Kansas, USA; 4 Higuchi Biosciences Center, University of Kansas, Lawrence, Kansas, USA

**Keywords:** molecular chaperone, protein aggregation, small-molecule inhibitor, antimicrobial compound, AAA+ ATPase, ClpB, DnaK, DBeQ, N2,N4-dibenzylquinazoline-2,4-diamine, DMSO, dimethyl sulfoxide, G6PDH, glucose-6-phosphate dehydrogenase, Hsps, heat-shock proteins, KJE, DnaK–DnaJ–GrpE, NBD, nucleotide-binding domain, SPR, surface plasmon resonance, TCEP, tris(2-carboxyethyl)phosphine

## Abstract

The ClpB–DnaK bichaperone system reactivates aggregated cellular proteins and is essential for survival of bacteria, fungi, protozoa, and plants under stress. AAA+ ATPase ClpB is a promising target for the development of antimicrobials because a loss of its activity is detrimental for survival of many pathogens and no apparent ClpB orthologs are found in metazoans. We investigated ClpB activity in the presence of several compounds that were previously described as inhibitor leads for the human AAA+ ATPase p97, an antitumor target. We discovered that N^2^,N^4^-dibenzylquinazoline-2,4-diamine (DBeQ), the least potent among the tested p97 inhibitors, binds to ClpB with a K_d_∼60 μM and inhibits the casein-activated, but not the basal, ATPase activity of ClpB with an IC_50_∼5 μM. The remaining p97 ligands, which displayed a higher affinity toward p97, did not affect the ClpB ATPase. DBeQ also interacted with DnaK with a K_d_∼100 μM and did not affect the DnaK ATPase but inhibited the DnaK chaperone activity *in vitro*. DBeQ inhibited the reactivation of aggregated proteins by the ClpB–DnaK bichaperone system *in vitro* with an IC_50_∼5 μM and suppressed the growth of cultured *Escherichia coli*. The DBeQ-induced loss of *E. coli* proliferation was exacerbated by heat shock but was nearly eliminated in a ClpB-deficient *E. coli* strain, which demonstrates a significant selectivity of DBeQ toward ClpB in cells. Our results provide chemical validation of ClpB as a target for developing novel antimicrobials. We identified DBeQ as a promising lead compound for structural optimization aimed at selective targeting of ClpB and/or DnaK.

On infection of a host, pathogens experience heat shock and oxidative stress, and their survival depends on molecular responses to these external conditions. The pathogen stress response has emerged as a critical mechanism for the development of novel antimicrobials (1, 2, 3). However, no successful inhibition of pathogen stress-response machinery has been developed to date, mostly due to sequence conservation among heat-shock proteins (Hsps) across different domains of life.

The Hsp100 family offers a unique opportunity for inhibitor development among Hsps. The ATP-dependent Hsp100 chaperones are essential for the survival of bacteria (where such proteins are called ClpB), lower eukaryotes (fungi and protozoa), and plants under stressful conditions. Unlike other heat-shock families, Hsp100 chaperones are not found in animals or humans (4, 5). Hsp100 chaperones, in cooperation with Hsp70 and Hsp40 (bacterial DnaK and DnaJ), are uniquely responsible for reactivation of aggregated proteins in microbial cells (6, 7, 8).

Hsp100 chaperones are required for invasiveness and/or in-host survival of multiple significant bacterial and protozoan pathogens including the ESKAPE bacteria (9, 10, 11), *Mycobacterium tuberculosis* (12, 13), *Mycoplasma pneumoniae* (14), *Salmonella typhimurium* (15), *Shigella dysenteriae* (16, 17), *Porphyromonas gingivalis* (18), *Francisella tularensis* (19), *Leptospira interrogans* (20), *Leishmania donovani* (21), and the tick-transmitted *rickettsia Ehrlichia chaffeensis* (22, 23). In the malaria parasite *Plasmodium falciparum*, there are two apparent Hsp100 isoforms and each one supports a different essential element of *Plasmodium* infectivity (24, 25, 26, 27). Thus, inhibition of Hsp100 may suppress infectivity and survival of a broad range of clinically relevant pathogenic microorganisms. Unfortunately, no high-affinity Hsp100-selective inhibitors are known.

Hsp100 chaperones are a subgroup of the AAA+ family of ATPases associated with different cellular activities (28). Like most AAA+ ATPases, Hsp100s form cylinder-shaped hexamers and use energy from ATP hydrolysis to induce conformational rearrangements in protein substrates (29). Hsp100-mediated reactivation of aggregates is linked to a forced extraction of polypeptides from aggregated particles and their subsequent unfolding by translocation through a narrow channel at the center of the Hsp100 hexamer (30, 31). Small-molecule inhibitors have been recently developed for the human AAA+ ATPase, p97, a promising antitumor target (32, 33, 34), which led to clinical evaluation and validated the drugability of AAA+ proteins. We reasoned that inhibitors of AAA+ ATPases distantly related to Hsp100 might serve as prototype scaffolds for the development of Hsp100-selective ligands. In this study, we investigated the effects of several p97 inhibitor leads on the activity of bacterial ClpB. We discovered that one of the known p97 inhibitors, N^2^,N^4^-dibenzylquinazoline-2,4-diamine (DBeQ), is a promising candidate compound for targeting Hsp100 chaperones.

## Results

### DBeQ inhibits the casein-activated ATPase activity of ClpB

**Figure 1:**
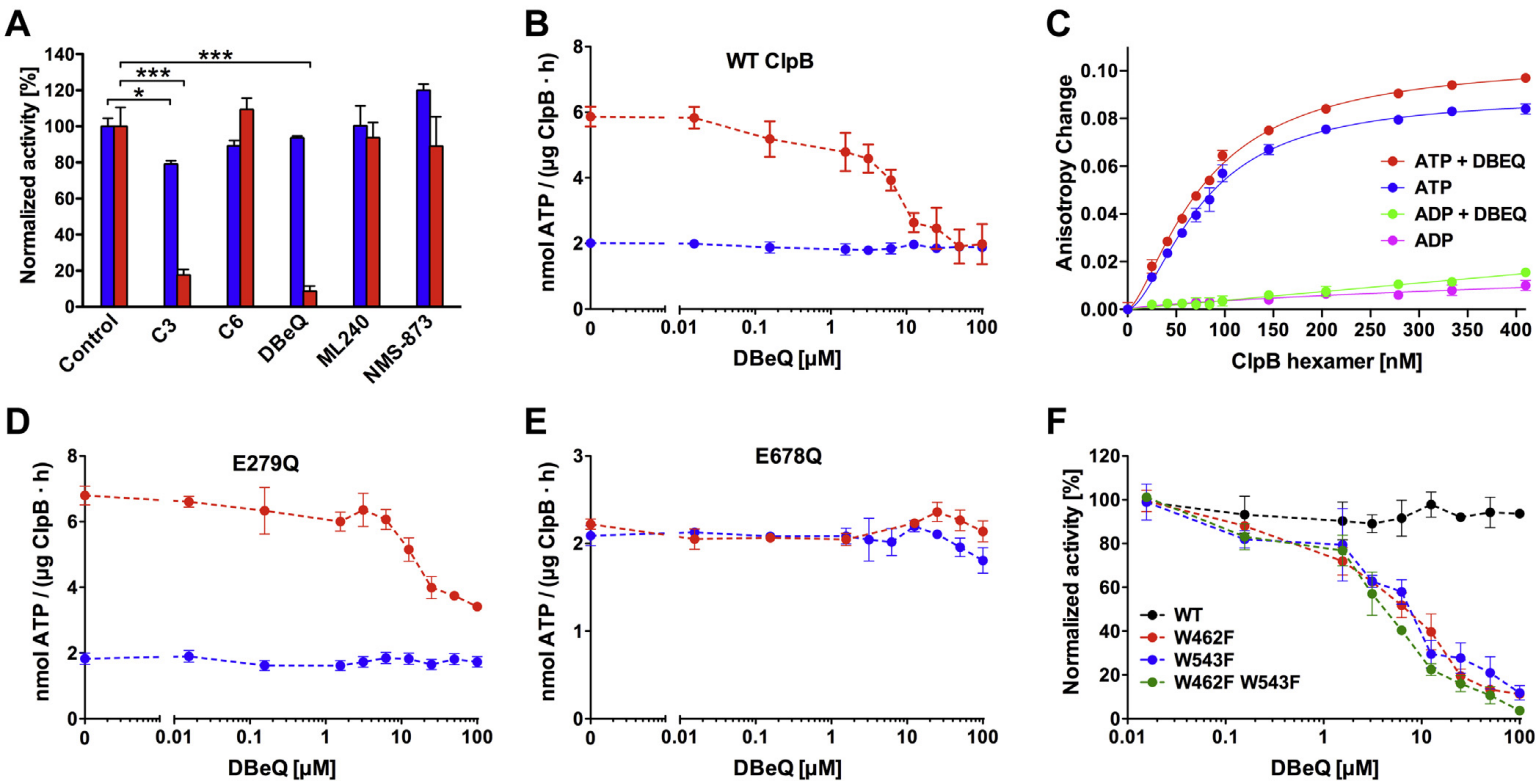
**Effects of the inhibitor candidates on the ATPase activity of ClpB at 37 °C.***A*, the ClpB ATPase activity was determined in the absence (*blue*) and presence (*red*) of the pseudosubstrate κ-casein without (control) and with 100-μM inhibitor candidates (see Fig. S1). The average activity values from three measurements normalized to the control are shown with SDs; ∗*p* < 0.05; ∗∗∗*p* < 0.001. *B*, *D*, *and E*, the ClpB ATPase activity was measured in the absence (*blue*) and presence (*red*) of κ-casein for wt ClpB (*B*), E279Q (*D*), and E678Q (*E*). *C*, FITC fluorescence anisotropy changes were determined on titrating ClpB E279Q/E678Q into 100-nM FITC-casein with 5-mM ATP or ADP and with or without 80-μM DBeQ. The average values from two independent measurements are shown with SDs. *Solid lines* show the results of fitting of the binding isotherm equation (see Experimental procedures), which gave the following values for ATP with DBeQ—K_d_, 78.5 nM; saturation signal, 0.11; Hill number, 1.48; r^2^, 0.997 and for ATP without DBeQ—K_d_, 77.3 nM; saturation signal, 0.09; Hill number, 1.68; r^2^, 0.994. *F*, a comparison of the basal ATPase activity of wt ClpB (*black*) with W462F (*red*), W543F (*blue*), and the double-mutant W462F/W543F (*green*). The average values from three measurements are shown with SDs. DBeQ, N^2^, N^4^-dibenzylquinazoline-2, 4-diamine.

We investigated the ATPase activity of *Escherichia coli* ClpB in the presence of three previously described p97 inhibitors: DBeQ (32), its p97-optimized derivative ML240 (35), and an alkylsulfanyl-1,2,4-triazole NMS-873 (36). We also tested two ClpB inhibitor candidates previously identified through a high-throughput ClpB interaction screen: C3 and C6 (37) (Fig. S1). None of the above compounds, except C3, showed a significant inhibition of the basal ClpB ATPase at 100 μM (Fig. 1*A*). However, only C3 and DBeQ strongly inhibited the ClpB ATPase in the presence of casein, a known pseudosubstrate of ClpB and an activator of the ClpB ATPase (Fig. 1, *A*–*B*). We also identified DBeQ, but not NMS-873, as a candidate ClpB ATPase inhibitor in the presence of casein in the pilot screen of 388 bioactive compounds (Fig. S2). We therefore focused the following studies on DBeQ as a novel ClpB inhibitor candidate.

The solubility of DBeQ in aqueous buffers is limited (32), but we found that the DBeQ absorbance obeys the Beer–Lambert law and the solution turbidity does not significantly increase for concentrations of up to 100-μM DBeQ in the presence of 1% dimethyl sulfoxide (DMSO) (Fig. S3). DBeQ suppressed the ClpB ATPase in the presence of casein to the basal level with an apparent IC_50_ ∼ 5 μM (Fig. 1*B*), but did not inhibit the ATP-dependent binding of casein to ClpB (Fig. 1*C*). We introduced E-to-Q substitutions in the Walker B motif in each of the two ATP-binding domains of ClpB (D1 and D2, see Fig. S4) to disable ATP hydrolysis activity in each domain. Of the two ATP-binding domains of ClpB, only the C-terminal one (*i.e.*, D2) showed casein-induced activation and was affected by DBeQ (Fig. 1, *D*–*E*).

ClpB contains two Trp residues, W543 located between D1 and D2 and W462 in the coiled-coil middle domain (see Fig. S4). Remarkably, the ClpB variants with each of the two Trp residues substituted with Phe became sensitive to inhibition by DBeQ even in the absence of casein (Fig. 1*F*). The sedimentation coefficient distributions for wt ClpB and the Trp/Phe variants (Fig. S5) showed a dominant ∼13 to 15 *S* molecular species, which corresponds to a hexameric ClpB in equilibrium with monomers (38). Importantly, no shifts of the sedimentation coefficient distribution maximum toward a lower s_20,w_ were observed in the presence of DBeQ (Fig. S5), which indicates that DBeQ does not induce dissociation of the ClpB hexamers. Overall, the different parameters tested above showed that DBeQ directly affects ClpB but does not compromise its structural integrity, with the basal ATPase unaffected by DBeQ even at 45 °C (Fig. S6).

### DBeQ inhibits the aggregate-reactivation activity of ClpB–DnaK

**Figure 2:**
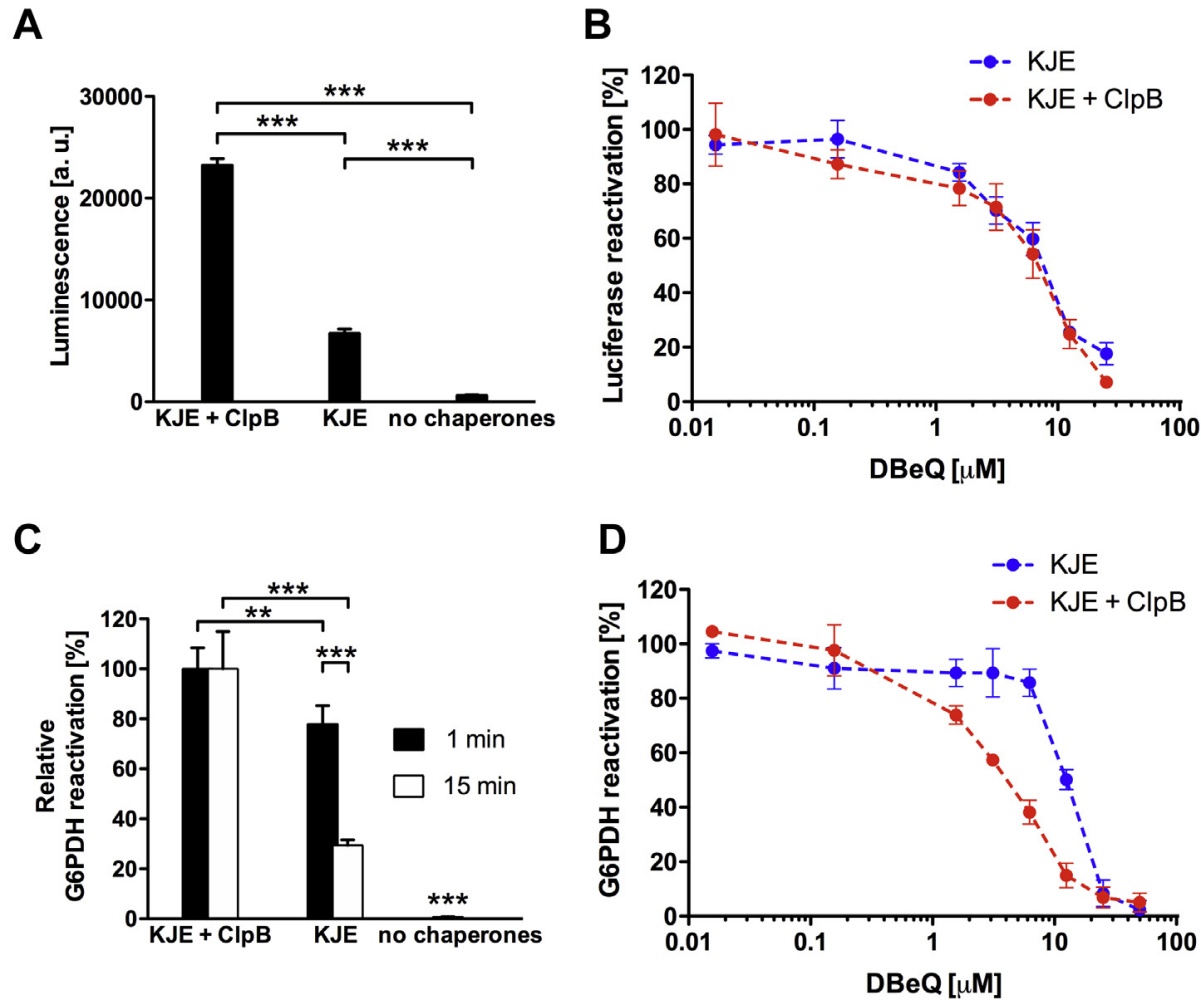
**Effects of DBeQ on the reactivation of protein aggregates mediated by ClpB and DnaK–DnaJ–GrpE (KJE).***A*, the activity of the thermally aggregated firefly luciferase after 20 min of reactivation at 25 °C in the presence of KJE, KJE + ClpB, or without the chaperones. The average values from three measurements are shown with SDs; ∗∗∗*p* < 0.001. *B*, the effects of DBeQ on the reactivation of aggregated luciferase in the presence of KJE (*blue*) or KJE + ClpB (*red*). The average values from three measurements are shown with SDs. *C*, the activity of the aggregated glucose-6-phosphate dehydrogenase (G6PDH) after 60 min of reactivation at 30 °C in the presence of KJE, KJE + ClpB, or without the chaperones. The aggregation of G6PDH was arrested after 1 min (*black bars*) or 15 min (*open bars*) to produce two samples with a different average size of aggregates. The average values from three measurements are shown with SDs; ∗∗∗*p* < 0.001; ∗∗*p* < 0.01. *D*, the effects of DBeQ on the reactivation of aggregated G6PDH. G6PDH samples aggregated for 1 min were used as substrates for KJE (*blue*). G6PDH samples aggregated for 15 min were used as substrates for KJE + ClpB (*red*). The average values from three measurements are shown with SDs. The data in panels *C* and *D* were normalized to the activity levels measured in the absence of DBeQ. DBeQ, N^2^, N^4^-dibenzylquinazoline-2, 4-diamine.

We tested the effect of DBeQ on the reactivation of aggregates prepared from two model substrates: firefly luciferase and bacterial glucose-6-phosphate dehydrogenase (G6PDH), whose native activity was not affected by DBeQ (Fig. S7). Either substrate can be reactivated by DnaK–DnaJ–GrpE (KJE), albeit with a significantly lower efficiency than by ClpB with KJE (Fig. 2, *A* and *C*), which allowed us to test the effect of DBeQ on both components of the ClpB–DnaK bichaperone system. Unexpectedly, we found that DBeQ inhibited both ClpB-dependent and ClpB-independent reactivation of aggregated luciferase with a similar IC_50_ value (Fig. 2*B*), which suggests that the rate of luciferase reactivation is limited by the activity of KJE. For aggregated G6PDH, however, ClpB-dependent reactivation was inhibited by DBeQ with a several-fold lower IC_50_ than ClpB-independent reactivation by KJE alone (Fig. 2*D*). These results indicate that DBeQ inhibits the activity of not only ClpB but also DnaK. However, we did not detect inhibition of the DnaK ATPase activity by DBeQ (Fig. S8*A*) or suppression of DnaK binding to a substrate-mimicking peptide (Fig. S8*B*).

### DBeQ interacts with ClpB and DnaK

**Figure 3:**
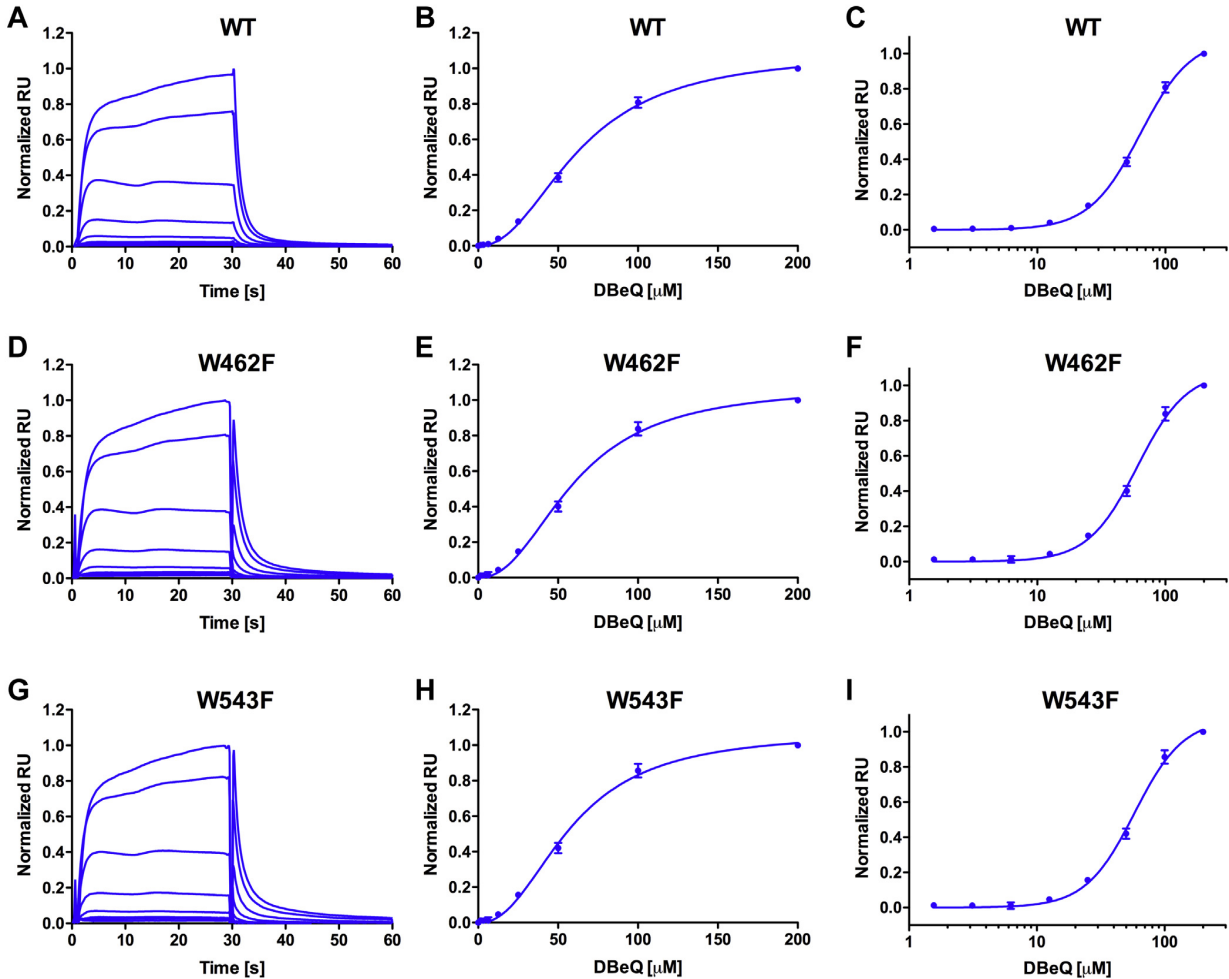
**Surface plasmon resonance (SPR) analysis of the binding of DBeQ to wt ClpB (A-C), W462F (D-F), and W543F (G-I).***A*, *D*, *and G*, representative SPR sensograms for three ClpB variants. Each *solid line* represents a sensogram obtained with a given DBeQ concentration; a higher DBeQ concentration produces a higher SPR response. DBeQ binding isotherms are shown with the linear ligand concentration scale (*B*, *E*, and *H*) or the logarithmic scale (*C*, *F*, and *I*). Shown are the averages with SDs from three repeated experiments. Solid lines in the panels *B*, *C*, *E*, *F*, *H*, and *I* represent the fits of the cooperative binding model with the parameters listed in Table S1. DBeQ, N^2^, N^4^-dibenzylquinazoline-2, 4-diamine.

We used surface plasmon resonance (SPR) to verify a direct interaction between DBeQ and ClpB (Fig. 3) or DnaK (Fig. S9). The binding isotherms for DBeQ and ClpB showed positive cooperativity (Fig. 3*B*) with an apparent K_d_ ∼ 60 μM and a Hill coefficient ∼2.3 (Table S1). The DBeQ binding isotherms were virtually identical for wt ClpB and its 2 Trp/Phe variants (Fig. 3). In contrast to ClpB, the DBeQ interaction with DnaK was noncooperative (Fig. S9, *A*–*C*) with an apparent K_d_ ∼ 100 μM (Table S1). The above apparent binding affinity of DnaK for DBeQ should be considered approximate, as it is close to the ligand solubility limit.

We determined that DBeQ interacted with the isolated nucleotide-binding domain (NBD) of DnaK with a similar affinity as with the full-length protein (Fig. S9, *D*–*F*, Table S1). As observed before (39), the ATPase activity of the DnaK NBD is similar to that of the full-length DnaK (Fig. S8*A*). We found that DBeQ did not inhibit the ATPase activity of the isolated NBD of DnaK (Fig. S8*A*).

### DBeQ suppresses the proliferation and survival of *E. coli*

**Figure 4:**
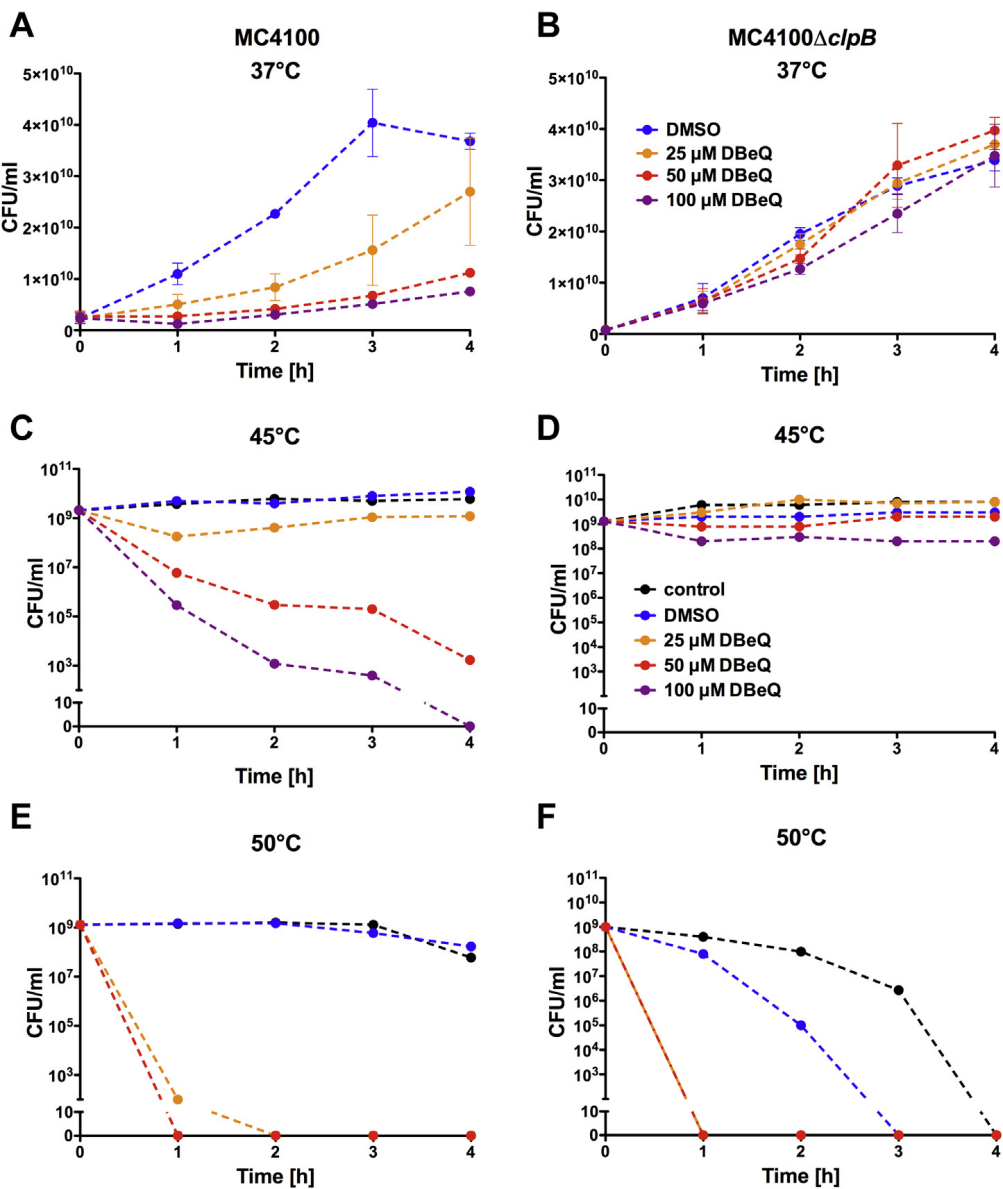
**Effects of DBeQ on the growth and viability of *E. coli* and its ClpB-deficient strain.** Growth of *E. coli* MC4100 (*A*) and MC4100*ΔclpB* (*B*) at 37 °C in the presence of an increasing concentration of DBeQ. Shown are the averages with SDs from three independent experiments. *C*–*F*, survival time courses for MC4100 (*C* and *E*) and MC4100*ΔclpB* (*D* and *F*) at 45 °C (*C* and *D*) and 50 °C (*E* and *F*) in the presence of an increasing concentration of DBeQ. Shown are representative results from 3 repeated experiments, including those in Figure 5. The legend in panel *B* applies also to panel *A*. The legend in panel *D* applies also to panels *C*, *E*, and *F*. DBeQ, N^2^, N^4^-dibenzylquinazoline-2, 4-diamine.

**Figure 5:**
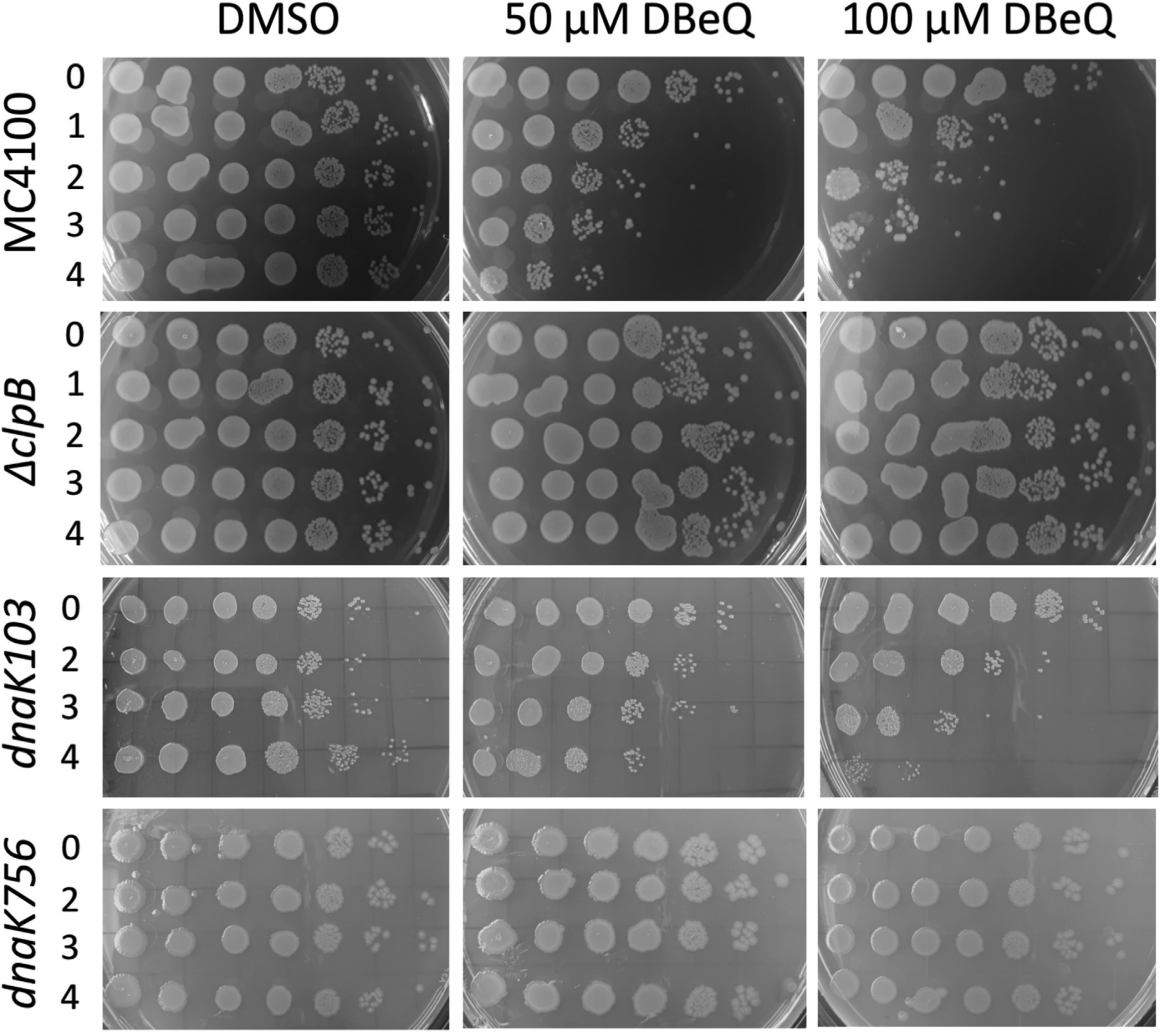
**Viability of *E. coli* strains after exposure to increasing concentrations of DBeQ at 45 °C.** The indicated strains of *E. coli* were incubated at 45 °C in the presence of DBeQ or with only DMSO for the period of time indicated on the left (in hours) and then spotted on agar plates and incubated overnight at 37 °C. Each spot on the agar plates represents a viable culture after a 10-fold serial dilution (from *left to right*). Shown are representative results from 3 repeated experiments. DBeQ, N^2^, N^4^-dibenzylquinazoline-2, 4-diamine. DMSO, dimethyl sulfoxide.

**Figure 6:**
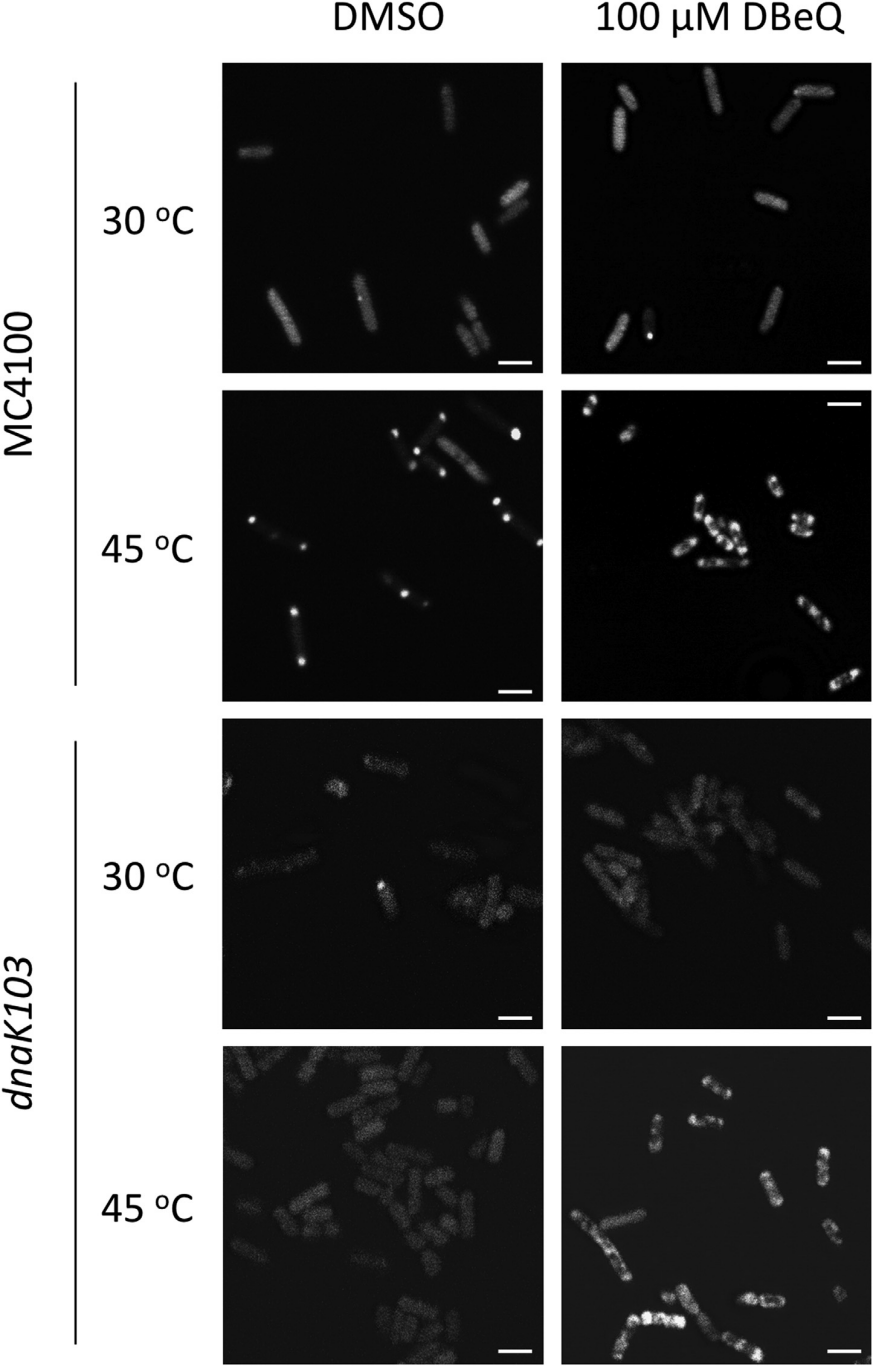
**Localization of ClpB-YFP in *E. coli*.** ClpB-YFP was expressed in the indicated *Escherichia coli* strains. The cells were grown at 30 °C and then shifted to 45 °C for 30 min (see Experimental Procedures). The images show YFP fluorescence signal. Representative images from 3 independent experiments are shown. The *white bar* in each panel corresponds to 2 μm.

ClpB is produced in *E. coli* cultured at 37 °C and is strongly upregulated during heat shock (Fig. S10). The Δ*clpB* strain, which does not produce ClpB (40), does not show growth defects at 37 °C or during a mild heat shock at 45 °C but rapidly loses viability at 50 °C (Fig. 4, *B*, *D* and F). DBeQ inhibited the growth of *E. coli* at 37 °C in a concentration-dependent manner (Fig. 4*A*), but the DBeQ effects were eliminated in the Δ*clpB* strain (Fig. 4*B*). The viability of *E. coli* at 45 °C (but not the Δ*clpB* strain) was strongly inhibited by DBeQ (Fig. 4, *C*–*D* and Fig. 5). On expression of a ClpB–YFP fusion protein in the Δ*clpB* strain (for the experiments shown in Fig. 6), the cells’ susceptibility to DBeQ was restored and was exacerbated at higher levels of ClpB expression (Fig. S11). A critical role of ClpB in *E. coli* survival under severe stress manifested at 50 °C, as shown by a loss of viability of the Δ*clpB* strain even without DBeQ (Fig. 4*F*). *E. coli* with an intact *clpB* gene survived the heat shock at 50 °C for several hours but lost viability at the lowest DBeQ concentrations tested (Fig. 4*E*). These experiments demonstrate that a treatment of *E. coli* with DBeQ is toxic to the cells, but the DBeQ toxicity at a moderate stress of 45 °C is more severe than that caused by a loss of ClpB in the Δ*clpB* strain.

To test the effects of DBeQ on the cross talk between ClpB and DnaK, we investigated the effects of DBeQ on viability of 2 *E. coli* strains with defective DnaK: *dnaK103*, which produces a truncated inactive DnaK that does not bind to its substrates (41, 42), and *dnaK756*, in which substrate release from DnaK is inefficient (43). We found that DBeQ suppressed viability of *dnaK103*, but surprisingly, viability of *dnaK756* was not affected by DBeQ (Fig. 5).

To further explore the effects of DBeQ on the function of ClpB in *E. coli* cells, we tracked the ClpB localization with confocal fluorescence microscopy using a ClpB construct with a C-terminally fused YFP. As demonstrated earlier (42, 44), heat-induced aggregates of thermosensitive proteins, such as *Photinus pyralis* luciferase, accumulate at the poles of *E. coli* cells (Fig. S12). ClpB and DnaK colocalize with the aggregates ((44) and Fig. 6). Importantly, the absence of a functional DnaK in the *dnaK103* strain prevents ClpB from accumulating with the aggregates (Fig. 6 and (42)), but in the presence of DBeQ, ClpB colocalizes with the aggregates even in *dnaK103* (Fig. 6). The heat shock–induced localization of ClpB at the cellular poles in the *dnaK756* strain with or without DBeQ was indistinguishable from that in wt *E. coli* (Fig. S13). The results with *dnaK103 E. coli* show that DBeQ affects localization of ClpB in cells exposed to heat shock. The fluorescent foci observed in *E. coli* in the presence of DBeQ are more diffuse than those found in the absence of the ligand, an effect exacerbated in the *dnaK103* strain (Fig. 6). Because the foci localization was attributed to nucleoid occlusion (44), an altered foci appearance in the presence of DBeQ may reflect a modified size of the nucleoid-free space in cells whose proliferation was challenged with DBeQ and/or a loss of a functional DnaK.

## Discussion

We hypothesized that small-molecule ligands of one AAA+ ATPase, p97, might also interact with another one, ClpB. We tested three inhibitors that show an increasing potency toward p97 (45): DBeQ (p97 IC_50_∼3 μM), ML240 (p97 IC_50_∼0.1 μM), and NMS-873 (p97 IC_50_∼0.02 μM). We discovered that among the three ligands, only DBeQ, the least potent one toward p97, affected ClpB (Fig. 1) with an apparent IC_50_ close to that displayed toward p97. ML240 is a p97-optimized DBeQ derivative, and its loss of potency toward ClpB suggests that a structural diversity among AAA+ ATPases is sufficient to allow discrimination between different ligands from the same chemical family. Thus, it may be feasible to orthogonally modify DBeQ and achieve an enhanced selectivity toward ClpB with a lower potency toward p97 and the other AAA+ ATPases.

Antimicrobial activity of 2,4-diaminoquinazolines has been reported before (46, 47), but without a clear identification of their cellular targets. We have now shown that ClpB is the main target of DBeQ in *E. coli* under both permissive conditions and during heat stress (Figs. 4 and 5). It is remarkable that the DBeQ-induced inhibition of *E. coli* viability depends on production of a single protein, ClpB. Although the Δ*clpB* strain does not respond to DBeQ, it becomes susceptible upon expression of ClpB (Fig. S11), which indicates that ClpB is required for the sensitivity of *E. coli* toward DBeQ. p97, the known DBeQ target, is not produced in bacteria. Interestingly, eukaryotic parasites, including *Plasmodium* and *Leishmania*, produce p97 as well as ClpB. An antiparasitic activity of DBeQ has been reported (48, 49), and it remains to be determined if the compound’s preferred *in vivo* target is p97 or ClpB.

As shown by SPR, DBeQ binds to ClpB with a positive cooperativity (Hill coefficient >2), which suggests multiple binding sites (Fig. 3 and Table S1). Detection of multiple binding sites for DBeQ is consistent with the oligomeric structure of ClpB. The apparent IC_50_ for DBeQ is an order of magnitude lower than the apparent K_d_ (Figure 1, Figure 2, Figure 3, and Table S1), which suggests that partial saturation of the DBeQ sites in the ClpB hexamer is sufficient for a strong inhibition. Indeed, it has been observed that incorporation of a single inactive subunit into a hexameric ClpB blocks aggregate reactivation (50).

ClpB-mediated reactivation of protein aggregates depends on several molecular processes: the ClpB hexamer assembly, ATP-dependent substrate binding, DnaK-dependent substrate engagement, and ATP hydrolysis–dependent substrate unfolding/translocation (4). Importantly, there is an allosteric linkage between substrate binding and the ATPase engine of ClpB, as demonstrated by activation of the ATPase in the presence of substrates (7). We found that DBeQ does not inhibit the ClpB hexamer assembly, substrate binding, or the basal ATPase of ClpB, but it does affect the linkage between substrate binding and the ATPase of the C-terminal D2 module (Fig. 1). Indeed, whereas substrate binding primarily occurs at the N-terminal region of ClpB (51, 52), a partial insertion of the substrate into the ClpB channel and its contact with the D2 module have been observed even without ATP hydrolysis (53). Moreover, D2 is fully responsible for the casein-induced activation of the ClpB ATPase because the activation is lost upon disabling D2 (Fig. 1*E*).

DBeQ-mediated modulation of the allostery within ClpB is further demonstrated by the apparent uncoupling of the DBeQ effects from substrate binding in the two Trp-to-Phe ClpB variants (Fig. 1*F*). Trp462 is located within the mobile coiled-coil domain of ClpB (Fig. S4), which controls the ATPase activity of the ClpB hexamer (54, 55). Trp543 is located at the interface between D1 and D2 (Fig. S4). Both Trp462 and Trp543 side chains are exposed at the intersubunit interface within the ClpB hexamer (Fig. S14). A similarity between the DBeQ binding isotherms for wt ClpB and its Trp/Phe variants (Fig. 3 and Table S1) indicates that the Trp/Phe substitutions do not produce additional DBeQ binding sites in ClpB. Altogether, the enhanced sensitivity of the Trp/Phe ClpB variants toward DBeQ suggests that the ligand binding site(s) may be located between the hexamer subunits and in the vicinity of the D1–D2 junction.

We found that aggregate reactivation with the ClpB–DnaK bichaperone system becomes inefficient in the presence of DBeQ (Fig. 2). Because the energy generated from ATP in the AAA+ engine is directly coupled with substrate translocation/unfolding (56, 57), our results suggest that DBeQ affects a linkage between the ClpB ATPase and substrate translocation by suppressing the acceleration of ATPase in response to a substrate, which decelerates substrate translocation and makes its reactivation inefficient.

An unexpected result of our study is a discovery of interactions between DBeQ and DnaK (Fig. S9) as well as inhibition of the DnaK-mediated reactivation of protein aggregates *in vitro* (Fig. 2). Unlike ClpB, DnaK is monomeric in solution and supports noncooperative binding of DBeQ (Fig. S9, Table S1). DBeQ binds to the NBD of DnaK (Fig. S9) but does not inhibit its ATPase (Fig. S8*A*). Our results are consistent with a recent report on the binding of amino-quinazolines within the ATP site of Hsp70 (58) but may also indicate a nonspecific interaction due to a predominance of hydrophobic groups in DBeQ. Interestingly, C3 also inhibits KJE (37) as well as ClpB and even luciferase (Fig. 1*A*, Fig. S7*A*). These results demonstrate a challenge in designing selective inhibitors for different families of molecular chaperones.

In *vitro* binding of DBeQ to DnaK notwithstanding, ClpB appears as the main target of DBeQ in *E. coli* because the compound’s effects manifest even in the absence of functional DnaK in the *dnaK103* strain but do not manifest in the Δ*clpB* strain (Fig. 5). Remarkably, at 45 °C, inhibition of ClpB with DBeQ is more detrimental for bacterial survival than a lack of ClpB in the Δ*clpB* strain. Thus, the phenotype of chemical inhibition of ClpB transcends a loss of function, at least under moderate stress.

Toxicity of ClpB in bacteria and its ortholog Hsp104 in yeast has been observed for “hyperactive” protein variants with mutations within the coiled-coil middle domain (55, 59, 60). However, unlike the DBeQ-treated ClpB, the toxic hyperactive variants display elevated ATPase and disaggregase activities.

The apparent toxic gain of function of the DBeQ-inhibited ClpB in bacterial cells could be blamed on DBeQ-induced aggregation of ClpB. However, we have not detected a propensity of ClpB to misfold and/or aggregate in the presence of DBeQ *in vitro* (Fig. S5), which is also supported by a lack of DBeQ effects on the basal ATPase activity of ClpB (Fig. 1 and Fig. S6). Moreover, the survival of *dnaK756 E. coli* upon treatment with DBeQ does not support a premise that the compound’s toxicity in cells is linked to misfolding or aggregation of its main target, ClpB, because, in such a case, a viability rescue should not be expected in a strain with a nonproductive DnaK chaperone (43).

Chaperone-deficient strains of *E. coli*, Δ*clpB*, and *dnaK103* survive moderate heat stress of 45 °C, thanks to other protein quality control factors: chaperones and proteases whose highly promiscuous and redundant activities are sufficient for maintaining proteostasis in those strains (Fig. 5). Because DBeQ apparently targets ClpB and no other chaperones in *E. coli*, the Δ*clpB* strain is therefore resistant to DBeQ under moderate stress conditions.

Physiologically, DnaK recruits ClpB to the aggregates and hands the substrates over to ClpB for disaggregation. In wt *E. coli* cells, a recruitment of ClpB to the aggregated proteins located at the cell poles strictly depends on DnaK (42). A striking polar localization of ClpB in *dnaK103* cells in the presence of DBeQ suggests that the compound allows ClpB to overcome a deficiency of its recruiter cochaperone (Fig. 6). However, ClpB interactions with protein aggregates become nonproductive in the presence of DBeQ (Fig. 2). A nonproductive binding of ClpB to protein aggregates may become dominant and could suppress viability of *E. coli* because it may hinder access of other protein quality control factors to their aggregated substrates and irreversibly disturb cellular proteostasis. The above explanation of the DBeQ-induced phenotype in *E. coli* is corroborated by an unexpected rescue of cellular viability in the *dnaK756* strain (Fig. 5). The DnaK756 variant binds to aggregates but cannot release them (43). Apparently, a substrate-trapping capability of DnaK756 counteracts the nonproductive interactions of ClpB with the aggregates in the presence of DBeQ and is sufficient to preserve cellular viability. Thus, the apparent gain-of-function effect of DBeQ may be due to disturbing the balance of cellular proteostasis by stimulating nonproductive interactions of ClpB that suppress effectiveness of the remaining chaperones. Importantly, regardless of its exact mode of function in bacterial cells, DBeQ is a potent and highly selective molecular probe that targets and disrupts protein quality control in *E. coli*.

Molecular chaperones have not been previously explored as targets for novel antimicrobials. In this work, we demonstrated that the AAA+ disaggregase ClpB can be selectively targeted with a small-molecule ligand in bacterial cells and that such a treatment could produce a loss of bacterial viability. This result is significant because mammalian cells do not contain orthologues of ClpB (5), whereas many pathogenic microorganisms require the ClpB activity for infectivity and survival. We have also shown that owing to a complexity of the cellular protein control machinery, understanding the mechanism of action of chemical inhibitors and the cellular phenotypes they produce requires multiple orthogonal biochemical and biological tests, such as those used in this study.

## Experimental procedures

### DNA constructs

The nucleotide sequence encoding the full-length *E. coli* DnaK (residues 1–638) was amplified from plasmid pTTQ19*dnaK*^*+*^ (61) using the following primers: DnaK-NcoI (5′-ATATACCATGGGTAAAATAATTGGTATCGACC-3′) and DnaK-XhoI (5′-TATATCTCGAGTTTTTTGTCTTTGACTTCTTC-3′), where the engineered restriction sites are underlined. The PCR product did not contain the *dnaK* STOP codon. Subsequently, the *dnaK* sequence was cloned into the *Nco*I/*Xho*I restriction site of pET28a. The STOP codon was introduced between the *dnaK* coding sequence and the C-terminal His-tag sequence in pET28a by site-directed mutagenesis with the following primers: DnaK_STOPf (5′-GTCAAAGACAAAAAATAAGAGCACCACCACCACCACC-3′) and DnaK_STOPr (5′-GGTGGTGGTGGTGGTGCTCTTATTTTTTGTCTTTGAC-3′), where the STOP codon is underlined. The final construct (plasmid pET28-DnaK) was verified by DNA sequencing and was used for production of DnaK.

The DNA sequence encoding the NBD of *E. coli* DnaK (residues 1–388) was PCR-amplified from plasmid pTTQ19*dnaK*^+^ (61) using the following primers: NBD-NcoI (5′-ATATACCATGGGTAAAATAATTGGTATCGACC-3′) and NBD-XhoI (5′-TATATCTCGAGGTCTTTTACGTCACCAGTCAGAAC-3′), where the engineered restriction sites are underlined. Subsequently, the NBD sequence was cloned into the NcoI/XhoI restriction site of pET28a. The construct (pET28-NBD1) was verified by DNA sequencing and used for production of the DnaK NBD.

The plasmids pHSG575-ClpB-YFP and pBAD-YFP-Luciferase (44) were provided by Bernd Bukau and Axel Mogk (ZMBH, University of Heidelberg, Germany).

### Proteins

*E. coli* ClpB and its variants E279Q, E678Q, E279Q/E678Q, W462F, W543F, and W462F/W543F were produced as described previously (52, 62, 63). To produce *E. coli* DnaK, the *E. coli* strain BL21(DE3) was transformed with pET28-DnaK. Single transformants were used to prepare an overnight culture in the LB media supplemented with kanamycin (50 μg/ml). On the following day, the overnight culture was diluted 50-fold in 1 l of LB with kanamycin (50 μg/ml). The culture was incubated at 30 °C. When absorbance at 600 nm reached 0.4, IPTG was added to the final concentration of 0.5 mM and the culture was incubated for 2 h at 30 °C with shaking at 200 RPM. Then, bacterial cells were collected by centrifugation (Beckman JA-14, 3000 relative centrifugal force, 15 min, 4 °C). The pellet was suspended in 20 ml of cold buffer KA (25-mM Hepes-KOH, pH 7.6, 50-mM KCl, 2.5-mM MgCl_2_, 1-mM EDTA, 10-mM β-mercaptoethanol, 5% glycerol) by vortexing. Cells were disrupted by sonication followed by centrifugation (rotor Beckman JA-20 11,500 RPM, 60 min, 4 °C). The supernatant was loaded on a Q Sepharose column (3.2 cm × 18 cm) equilibrated with buffer KA. After washing with buffer KA (150 ml), proteins were eluted with an increasing gradient of buffer KB (25-mM Hepes-KOH, pH 7.6, 550-mM KCl, 2.5-mM MgCl_2_, 1-mM EDTA, 10-mM β-mercaptoethanol, 5% glycerol). The collected fractions, which contained DnaK, were pooled and then diluted 10-fold in buffer KC (50-mM Tris-HCl, pH 7.8, 1-mM EDTA, 5-mM β-mercaptoethanol, 10% glycerol). The sample was loaded on a DEAE-Sephacel column (3.2 × 12 cm) equilibrated with buffer KC. After washing the column with 100 ml of buffer KD (50 mM Tris-HCl pH 7.8, 50 mM NaCl, 1 mM EDTA, 5 mM β-mercaptoethanol, 5% glycerol), proteins were eluted with an increasing gradient of buffer KE (50-mM Tris-HCl, pH 7.8, 400-mM NaCl, 1-mM EDTA, 5-mM β-mercaptoethanol, 5% glycerol). The collected fractions were concentrated with a centrifugal filter device (10000 molecular weight cut-off, 3500 RPM, 4 °C). Concentrated samples were resolved on a Superdex 200 gel filtration column equilibrated with buffer KA. Fractions containing DnaK with purity greater than 95% were collected and concentrated to ∼2 mg/ml with a centrifugal filter device. Protein concentration was determined by the Bradford method. The aliquots of DnaK were stored at −20 °C.

To produce the DnaK NBD, *E. coli* strain BL21(DE3) was transformed with pET28-NBD1. Single transformants were used to prepare overnight culture in the LB media supplemented with kanamycin (50 μg/ml). The overnight culture was diluted 50-fold in 1 l of LB with kanamycin and incubated at 30°C. When absorbance at 600 nm reached 0.4, IPTG was added to the final concentration of 1 mM and the culture was incubated for 3 h at 30 °C with shaking at 200 RPM. The bacterial cells were collected by centrifugation (Beckman JA-14, 3000 relative centrifugal force, 15 min, 4 °C). The pellet was suspended in 20 ml of cold T10 buffer (40-mM Tris-HCl, pH 8.0, 100 mM KCl, 10 mM imidazole) by vortexing. Cells were disrupted by sonication. The soluble fraction was obtained by centrifugation (Beckman JA-20, 11,500 RPM, 60 min, 4 °C). The supernatant was loaded on a column with 5 ml of nickel-nitrilotriacetic acid resin equilibrated with buffer T10. The column was washed with 50 ml of buffer T20 (40-mM Tris-HCl, pH 8.0, 100-mM KCl, 20-mM imidazole). Proteins were eluted with buffer T250 (40-mM Tris-HCl, pH 8.0, 100-mM KCl, 250-mM imidazole). Fractions containing the NBD were pooled and concentrated with a centrifugal filter device (10000 molecular weight cut-off, 3500 RPM, 4 °C). The concentrated NBD sample was resolved on a Superdex 200 gel filtration column equilibrated with buffer B (50-mM Tris-HCl, pH 7.5, 0.2 M KCl, 20-mM MgCl_2_, 10% glycerol, 1-mM EDTA, 1-mM DTT). Fractions containing the NBD with purity greater than 95% were collected and concentrated to ∼5 mg/ml. Protein concentration was determined by the Bradford method. The aliquots of the NBD were stored at −20 °C.

Firefly luciferase, G6PDH from *Leuconostoc mesenteroides*, DnaJ, and GrpE were obtained as described before (25, 52, 62, 64). Peptide B2 and its FITC-labeled variant were obtained as described before (65). κ-Casein and FITC–casein were obtained from Sigma-Aldrich (St Louis, MO).

### Bacterial strains

The following strains of *E. coli* were used: MC4100, MC4100*ΔclpB::kan* (40), *dnaK*103 (41), and *dnaK*756 (66).

### Chemicals

Candidate inhibitor compounds DBeQ, ML240, and NMS-873 were purchased from Sigma-Aldrich (St Louis, MO) and used directly without further purification; C3 and C6 were obtained from TimTec (Newark, DE). The compounds in a powder form were dissolved in DMSO at 10 mM and used in biochemical studies after further dilutions.

### ATPase activity assays

To determine the ATPase activity, ClpB was diluted to 28 μg/ml (49-nM hexamer) in buffer C (50-mM Tris-HCl, pH 7.4, 20-mM MgCl_2_, 1-mM EDTA, 0.5-mM tris(2-carboxyethyl)phosphine [TCEP]). In some experiments, the ClpB solution was supplemented with 17.4 μg/ml (10 μM) κ-casein. The ClpB aliquots (18 μl) were mixed with 1 μl of the investigated compounds at different concentrations in 100% DMSO or with DMSO as a control. A sample without ClpB was used as a baseline control. After a 10-min preincubation at 37 or 45 °C, the ATP hydrolysis reaction was initiated by adding 1 μl of 100-mM ATP. The samples were incubated for 60 min (basal ClpB activity in the absence of κ-casein) or 15 min (in the presence of κ-casein) at 37 °C or 30 min at 45 °C in the absence of casein. For the E678Q ClpB variant, the incubation time in the presence of κ-casein was 60 min. After incubation, 15 μl of each sample was mixed with 200 μl of the ammonium molybdate/malachite green reagent (67) dispensed into a 96-well plate, followed by an addition of 30 μl of 34% sodium citrate (68). The plate was agitated inside a Synergy H1 reader (BioTek, Winooski, VT) for 15 min at room temperature, and the samples’ absorbance was measured at 630 nm. The readouts for ClpB-containing samples were corrected for absorbance of samples without ClpB, to account for nonenzymatic production of inorganic phosphate. A standard curve obtained with different inorganic phosphate concentrations was used to determine the amount of phosphate produced from ATP in the presence of ClpB.

To determine the ATPase activity of DnaK, 18 μl of DnaK solution or DnaK NBD solution prepared in buffer C was mixed with 1 μl of the investigated compounds or DMSO as a control. Samples without DnaK were used as control blanks. The samples were preincubated for 10 min at 37 °C. The reaction was initiated by adding 1 μl of 100-mM ATP bringing the final concentration of DnaK to 1.1 μM or DnaK NBD to 3.4 μM. The samples were incubated at 37 °C for 70 min for DnaK or 75 min for NBD. The concentration of inorganic phosphate produced from ATP in the presence of DnaK was determined as described above for ClpB.

### Aggregate reactivation assays

G6PDH was chemically denatured by mixing 5 μl of the G6PDH stock solution (441 μM) with 5 μl of buffer A (10 M urea, 16% glycerol, 40-mM DTT) preheated at 47 °C. The samples of G6PDH in buffer A were incubated for 5 min at 47 °C. Subsequently, 5 μl of denatured G6PDH was mixed with 95 μl of buffer B (50-mM Tris-HCl, pH 7.4, 20-mM Mg(OAc)_2_, 30-mM KCl, 1-mM EDTA, 1-mM β-mercaptoethanol) preheated at 47 °C to initiate protein refolding, which, under these conditions, is inefficient and leads to misfolding and aggregation of G6PDH (69). G6PDH aggregate production was arrested after 1 min (for refolding by KJE) or 15 min (for refolding by KJE with ClpB) by transferring into ice for 2 min. To observe the aggregate reactivation, aggregated G6PDH was diluted 10-fold in buffer C (50-mM Tris-HCl, pH 7.4, 20-mM MgCl_2_, 1-mM EDTA, 0.5-mM TCEP) containing the chaperones KJE (5.5-μM DnaK, 1.1-μM DnaJ, 1.1-μM GrpE) or KJE-ClpB (5.5-μM DnaK, 1.1-μM DnaJ, 1.1-μM GrpE, 1.65-μM ClpB (hexamer)). G6PDH in buffer C without chaperones was used as a control for a spontaneous aggregate reactivation. The chaperone-G6PDH samples (18 μl) were supplemented with 1 μl of DBeQ in DMSO or pure DMSO as a control. The samples were incubated for 2 min at 30 °C. The aggregate reactivation was initiated by adding 1 μl of 100-mM ATP, followed by incubation for 60 min at 30 °C. To determine the enzymatic activity of G6PDH, a 5-μl aliquot from the reactivated sample was mixed with 195 μl of buffer D (50-mM Tris-HCl, pH 7.8, 5-mM MgCl_2_, 2-mM glucose-6-phosphate, 1-mM NADP^+^) in a 96-well plate that had been preheated at 30 °C. The plate was inserted into Synergy H1 plate reader (BioTek, Winooski, VT) with the chamber temperature at 30 °C, and the sample absorbance at 340 nm was measured after 10 min. The amount of NADPH produced was calculated based on the NADPH molar extinction coefficient, Ɛ_340_ = 6220 [M^−1^ ⋅ cm^−1^].

Firefly luciferase (220 μM) was diluted 300-fold in PBS buffer with 1 mg/ml bovine serum albumin and then incubated for 12 min at 45 °C. The chaperone solutions were prepared in buffer C (50-mM Tris-HCl, pH 7.4, 20-mM MgCl_2_, 1-mM EDTA, 0.5-mM TCEP): KJE (1.15-μM DnaK, 1.15-μM DnaJ, 0.57-μM GrpE) and KJE-ClpB (1.15-μM DnaK, 1.15-μM DnaJ, 0.57-μM GrpE, 1.72-μM ClpB (hexamer)). Then, 17 μl of a chaperone solution was mixed with 1 μl of DBeQ in DMSO or pure DMSO as a control and preincubated for 10 min at 25 °C. Next, 1 μl of the heat-denatured luciferase (0.7 μM) and 1 μl of ATP (100 mM) were added to the samples, followed by incubation for 20 min at 25 °C. To determine the luciferase activity, 5 μl of each sample was transferred into 100 μl of the luciferase substrate (Promega), previously dispensed into a white 96-well plate. Luminescence was measured using Synergy H1 plate reader (BioTek, Winooski, VT).

### Surface plasmon resonance

ClpB, DnaK, and the NBD of DnaK were extensively dialyzed against the SPR buffer (20-mM Hepes, pH 7.4, 150-mM NaCl, 20-mM MgCl_2_, 1-mM EDTA, 1-mM DTT). After dialysis, the protein concentration was determined by the Bradford method. The DBeQ–protein interactions were studied using a Biacore T200 instrument (GE Healthcare) at 25 °C. The proteins were diluted to 300 μg/ml in 10-mM sodium acetate buffer (pH 5 for ClpB DnaK or pH 4 for NBD) and immobilized on HC1500M sensor chips (Xantec) by standard amine coupling chemistry as described previously (70). A reference flow cell was created by ethyl(dimethylaminopropyl) carbodiimide/N-hydroxysuccinimide activation followed by quenching with 1 M ethanolamine (pH 9.0). Solvent correction curves were obtained at the beginning and end and after every 50 injection cycles by injecting varying DMSO concentrations (4.0, 4.4, 4.6, 4.8, 5.0, 5.2, 5.4, and 5.9% [v/v]). All experiments were performed using a flow rate of 30 μl/min.

Binding isotherms were obtained with the ligand diluted to different concentrations in the SPR running buffer HBSMTD (20-mM Hepes, pH 7.4, 140-mM NaCl, 20-mM MgCl_2_, 5% (v/v) DMSO, 0.005% (v/v) Tween-20). Replicate injections were performed for all samples, and a buffer-only injection was performed for every 12 injection cycles. To test the structural integrity of ClpB after immobilization on the sensor chips, we performed control titrations with ATPγS and adenosine. The dissociation constants determined from those experiments were ∼20 μM for ATPγS and ∼2 mM for adenosine. These preliminary experiments indicated that the immobilization on the SPR chip preserved the oligomeric structure of ClpB because monomeric ClpB has low affinity toward ATP (K_d_ > 1 mM (62)). Moreover, the immobilized ClpB discriminated between a nucleotide analog and a nucleoside, which indicated an adequate structural integrity of ClpB on the sensor chips.

The resulting sensorgrams were analyzed using Biacore T200 Evaluation Software v3.0 according to the following procedure. Solvent correction curves were generated and applied to all data sets, and all sensorgrams were double-referenced by subtracting the most recent buffer blank injection. The signal immediately before injection stop of these corrected sensorgrams was treated as the binding response. GraphPad Prism was used for nonlinear least-squares fitting of the model assuming either noncooperative or cooperative binding of DBeQ to DnaK and ClpB, respectively.

### Bacterial viability

*E. coli* MC4100, MC4100Δ*clpB*, *dnaK*103, and *dnaK*756 strains were used to determine an influence of DBeQ on bacterial growth and viability. Bacteria were maintained in the LB media, and in the case of the MC4100Δ*clpB* strain, LB was supplemented with 30 μg/ml kanamycin. All experiments were initiated by preparing overnight cultures inoculated from single colonies and grown at 37 °C. The *dnaK*103 and *dnaK*756 overnight cultures were grown at 30 °C. On the following day, the cultures were diluted 100-fold in 10 ml of LB without antibiotics and incubated at 37 °C. The culture optical density was monitored at 600 nm. When absorbance at 600 nm reached 0.4, the culture was divided into 1-ml aliquots and supplemented with 10 μl of DMSO or different concentrations of DBeQ in DMSO. The samples were immediately transferred into an incubator/shaker at temperature 37, 45, or 50 °C. Bacteria were cultured for up to 4 h with shaking (200 RPM). At specific time points, 100 μl of each culture was withdrawn and serially diluted in sterile 0.9% NaCl up to 10^6^-fold dilution. To estimate the number of viable cells, 5 μl or 10 μl of the diluted cultures was spread on the LB-agar plate without an antibiotic. After a complete adsorption of liquid on the LB-agar surface, the plates were incubated overnight at 37 °C. On the following day, the bacterial colonies were counted and the colony-forming units/ml were calculated.

### Chemical library screening

An absorbance-based assay for ClpB ATPase activity was developed by using BioMol Green Reagent (Enzo Life Sciences, Farmington, NY) for screening in a 384-microplate–based format. The optimized assay conditions were as follows: ClpB (150 nM), ATP (200 mM), and κ-casein (25 μM) in 100-mM Tris/HCl, pH 8, 10-mM MgCl_2_, 1-mM DTT, and 1-mM EDTA. The samples were incubated for 10 min at 37 °C, and the ATP hydrolysis reaction was terminated by adding 80 μl of BIOMOL Green. Absorbance at 620 nm was measured with a PerkinElmer EnSpire. The optimized assay was used to screen compounds drawn from Selleck Bioactive library (1900 compounds; Selleck Chemicals, Houston, TX) in the presence or absence of κ-casein.

### Analytical ultracentrifugation

Protein samples (0.5 ml) of wt ClpB and its variants W462F and W543F were dialyzed against 3 changes of 1 l of buffer AUC (50-mM Tris-HCl, pH 7.4, 200-mM KCl, 20-mM MgCl_2_, 1-mM EDTA, 0.5-mM TCEP). After dialysis, the samples were centrifuged (15 min, 13,000 RPM, 4 °C). The supernatants were collected, and the protein concentration was determined with the Bradford assay. Sedimentation velocity experiments were performed in Beckman Optima XL-I analytical ultracentrifuge equipped with a 4-cell rotor and 2-sector cells. The ClpB samples were diluted to 1 mg/ml in the AUC buffer supplemented with DMSO (5% (v/v) final concentration) with 1-mM ATPɤS, with or without 50-μM DBeQ. Centrifugation was performed at 48,000 RPM at 20 °C. The sedimentation profiles were collected using the interference detection system and analyzed with the Origin software supplied by the instrument manufacturer using the time-derivative method of Stafford (71). Observed sedimentation coefficients were corrected to values corresponding to the density and viscosity of water (s_20,w_) using Sednterp software (www.jphilo.mailway.com) with additional corrections for the presence of 5% DMSO (72).

### Fluorescence anisotropy

To monitor interactions between casein and ClpB, we used FITC-casein and the substrate-trapping ClpB E279Q/E678Q variant (52, 73). The titration of FITC-casein with ClpB E279Q/E678Q in the presence of ATP or ADP and the fluorescence anisotropy measurements were performed as previously described (65).

To monitor interactions between the peptide B2 and DnaK, the DnaK stock was diluted in buffer C (50-mM Tris-HCl, pH 7.4, 20-mM MgCl_2_, 1-mM EDTA, 0.5-mM TCEP) to a final concentration of 1 μM. DBeQ solutions in DMSO (20 μl) were mixed with 372 μl of 1-μM DnaK. After 10 min of incubation at room temperature, the samples were supplemented with 8 μl of 1-μM FITC-B2 in buffer C (FITC-Ahx-QRKLFFNLRKTKQRLGWFNQ-NH_2_) (51). The FITC-B2 sample without DnaK was used as a control. After a 30-min incubation at room temperature, fluorescence anisotropy of FITC-B2 was measured with a PerkinElmer LS55 (λ_exc_ = 485 nm (slit: 15 nm), λ_em_ = 521 (slit: 20 nm)).

### Western blotting

The ClpB protein level in *E. coli* cells was analyzed by Western blotting. Overnight culture of each strain was diluted 100-fold and incubated at 37 °C with shaking (200 RPM). When the absorbance at 600 nm reached 0.4, the culture was split into two: for one, the incubation at 37 °C continued, while the second one was transferred to 45 °C. After 2 h of incubation at the target temperatures, the cells were collected by centrifugation. The supernatant was discarded, and cell pellets were suspended in 160 μl of 2x Laemmli sample buffer. Sixty microliters of each sample was resolved in duplicates in 8% SDS-PAGE gel. The gel was cut into two parts, one part was used for blotting onto a nitrocellulose membrane and the second part of gel was stained in Coomassie R-250. The membrane was blocked with 5% nonfat dried milk dissolved in TBST buffer (19-mM Tris-HCl, pH 7.4, 137-mM NaCl, 2.7-mM KCl, 0.2% Tween-20) overnight at 4 °C. Next, the membrane was incubated with rabbit polyclonal anti-ClpB IgG (1:50,000 in milk) for 1 h at room temperature. After incubation with the primary antibody, the membrane was washed in TBST buffer (4 times, 10 min). Next, the membrane was incubated with anti-rabbit horseradish peroxidase-conjugated secondary antibodies for 1 h at room temperature and washed in TBST (4 times, 10 min) and then TBST without Tween-20 (10 min). Signal detection was performed using SuperSignal West Pico Chemiluminescent Substrate (Pierce) and an Azure c500 digital imaging system (Azure Biosystems, Dublin, CA).

### Confocal microscopy

ClpB-YFP and Luciferase-YFP were expressed at low levels in *E. coli* strains Δ*clpB*, *dnaK*103, and *dnaK*756 as described before (44). The expression of ClpB-YFP and YFP-Luciferase was initiated by addition of 200-μM IPTG or 0.02% (w/v) arabinose, respectively. The bacterial cells were cultured at 30 °C with 100-μM DBeQ or DMSO only. Protein translation was stopped by addition of erythromycin (30 μg/ml), and each culture was divided into two samples. One sample remained at 30 °C (control), whereas the other sample was transferred to 45 °C for 30 min (heat-shock conditions).

A thin layer of 1% agarose (TopVision Low Melting Point Agarose, Thermo Scientific) was prepared on a clean microscope glass slide. A small drop of 1% agarose was placed near the frosted end of the glass slide while bringing a second glass slide at a 30 to 45° angle from above, allowing the agarose drop to spread along the contact edge. The top slide was moved gently across the bottom slide to produce an evenly spread thin layer of agarose. An *E. coli* culture sample (1 μl) was immediately placed on the agarose-covered glass slide, covered with a cover slip, and used for fluorescence imaging.

Fluorescence imaging was performed using a Carl Zeiss LSM 880 Airyscan confocal microscope equipped with a Plan-Apochromat 63x/1.40 Oil DIC M27 objective coupled with a YFP Filter (excitation at 514 nm, emission at 561 nm, laser strength 0.98%). Zen 2.3 lite software was used for image capture and processing.

## Data availability

All relevant data are available within this manuscript and the associated Supporting Information.

## Conflict of interest

The authors declare that they have no conflicts of interest with the contents of this article.
