# Supplementary material for: Repurposing p97 inhibitors for chemical modulation of the bacterial ClpB–DnaK bichaperone system
Source: J Biol Chem. 2020 Nov 21;296:100079. doi: 10.1074/jbc.RA120.015413 (PMC7948422; doi:10.1074/jbc.RA120.015413)
Supplement: Supplementary Figures and Tables [file mmc1.pdf]

Supporting Information for the article:

Repurposing p97 inhibitors for chemical modulation of the bacterial ClpB/DnaK bi-chaperone system

Przemyslaw Glaza, Chathurange B. Ranaweera, Sunitha Shiva, Anuradha Roy, Brian V. Geisbrecht, Frank J. Schoenen, and Michal Zolkiewski

Contents:

Supplementary Table 1

Supplementary Figures 1-14

Supplementary Table 1. **Thermodynamic parameters for the interaction between DBeQ and ClpB or DnaK.** The values were obtained by fitting a binding isotherm equation with positive cooperativity to the SPR data for ClpB and a non-cooperative binding isotherm equation to the SPR data for DnaK. B_{max} indicates the SPR signal at saturation, r is the correlation coefficient, r^2 represents the goodness of the fit.

**Protein**	**K_d [μM]**	**Hill coefficient**	**B_{max}**	**r^2**
ClpB	64.4 ± 4.7	2.3 ± 0.3	1.09 ± 0.02	0.999
ClpB W462F	61.4 ± 4.7	2.4 ± 0.4	1.08 ± 0.03	0.998
ClpB W543F	59.1 ± 4.1	2.4 ± 0.4	1.07 ± 0.03	0.998
DnaK	105 ± 17	-	2.07 ± 0.20	0.996
DnaK NBD	95 ± 16	-	1.98 ± 0.20	0.995

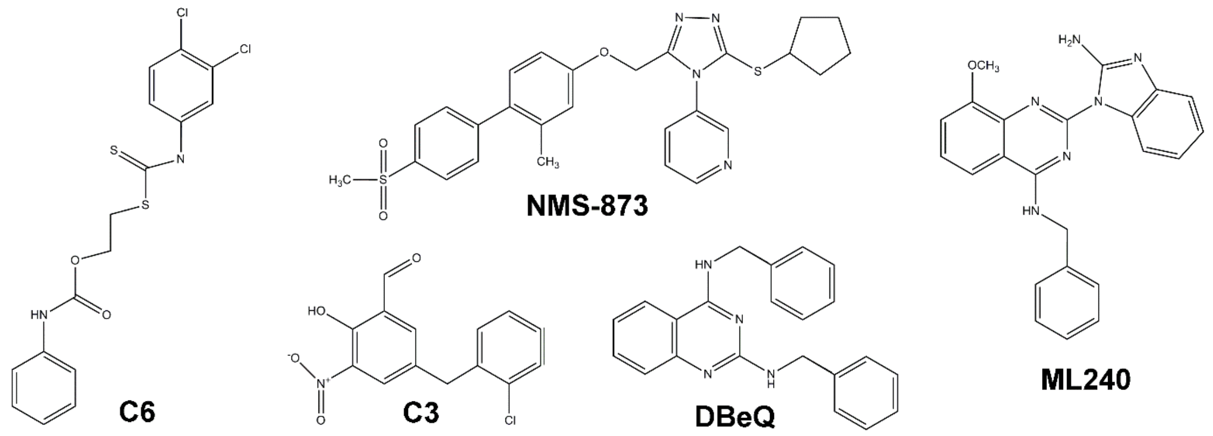

Supplementary Figure 1. **Chemical structure of the compounds investigated in this study** (based on the structures supplied by the manufacturer and redrawn using ChemDraw 12).

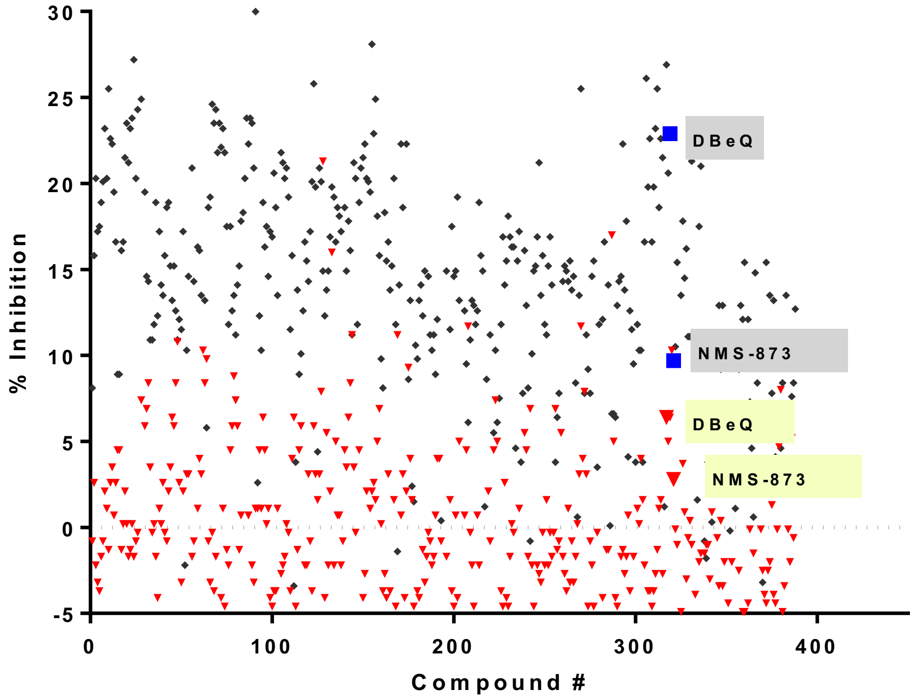

Supplementary Figure 2. Pilot screen for inhibitors of the ClpB ATPase activity. Shown is a scatter-graph of the ClpB ATPase inhibition across 388 compounds from Selleck Bioactive library screened in the presence (black) or absence (red) of 25 µM κ-casein.

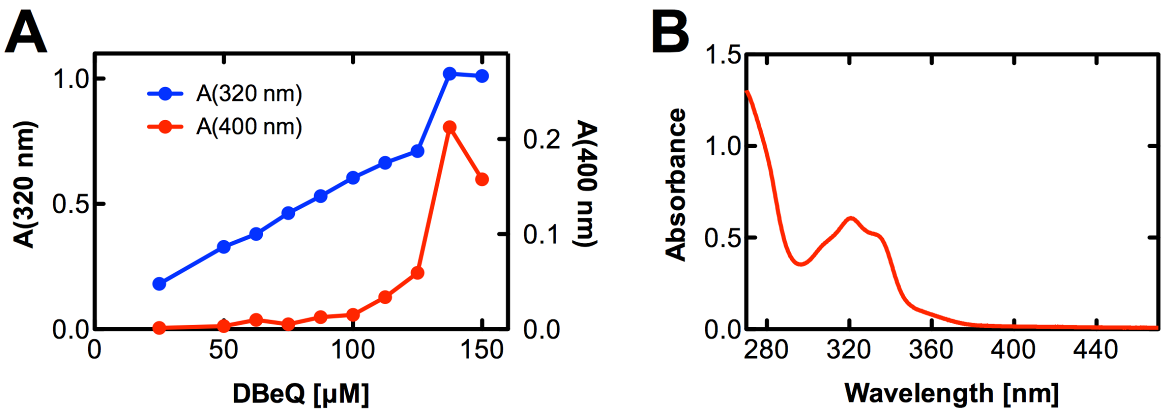

Supplementary Figure 3. Solubility of DBeQ in aqueous solution. Solutions of DBeQ were prepared in the ATPase assay buffer containing 1% DMSO. (A) DBeQ absorbance at 320 nm (blue) and solution turbidity at 400 nm (red) for increasing DBeQ concentrations. (B) UV absorption spectrum of 100 μM DBeQ.

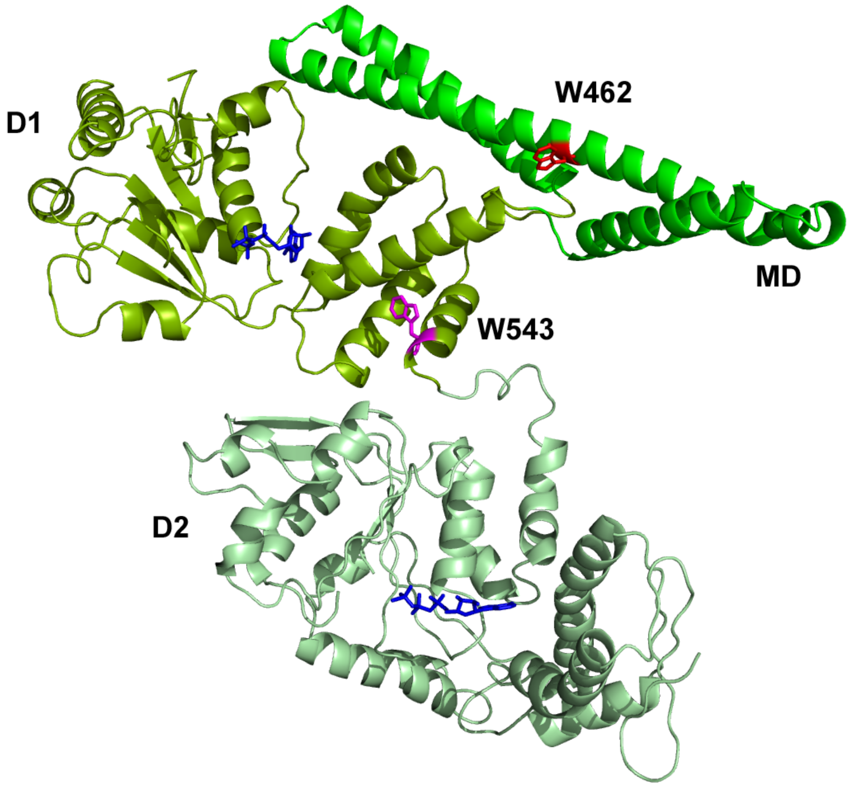

Supplementary Figure 4. **The ATP-binding modules (D1 and D2) separated with the middle domain (MD) in *E. coli* ClpB (PDB entry: 5OG1).** Trp462 (red), Trp543 (magenta), and the ATP-analog, ATPγS (dark blue) bound in D1 and D2 are shown with a "stick" representation. Image generated using PyMOL 1.3 (Schrödinger LLC, www.pymol.com).

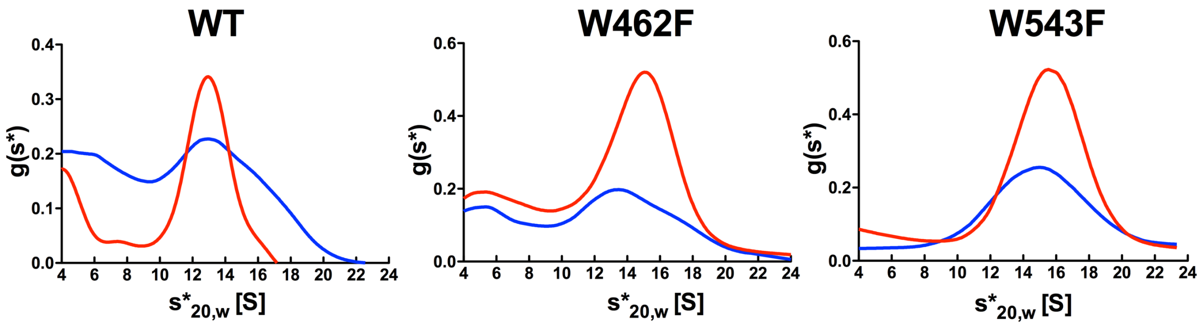

Supplementary Figure 5. **Sedimentation velocity analysis of wt ClpB, W462F, and W543F in the absence (blue) and presence (red) of 50 µM DBeQ.** Analytical ultracentrifugation was performed with 1 mg/ml ClpB in the presence of 1 mM ATPγS. Shown are the apparent distributions of sedimentation coefficient expressed in Svedbergs (S).

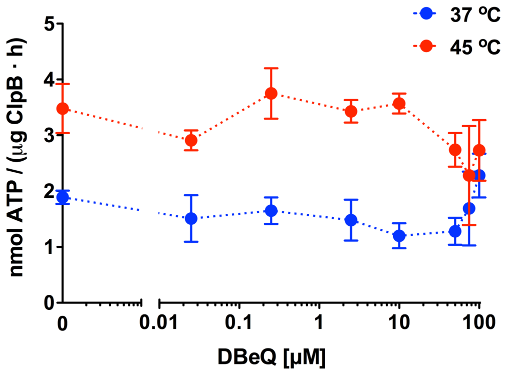

Supplementary Figure 6. DBeQ does not inhibit the ClpB ATPase under heat-shock conditions. The ATPase activity of wt ClpB was determined at 37 and 45 °C as the function of DBeQ concentration. Average values from 3 measurements are shown with standard deviations.

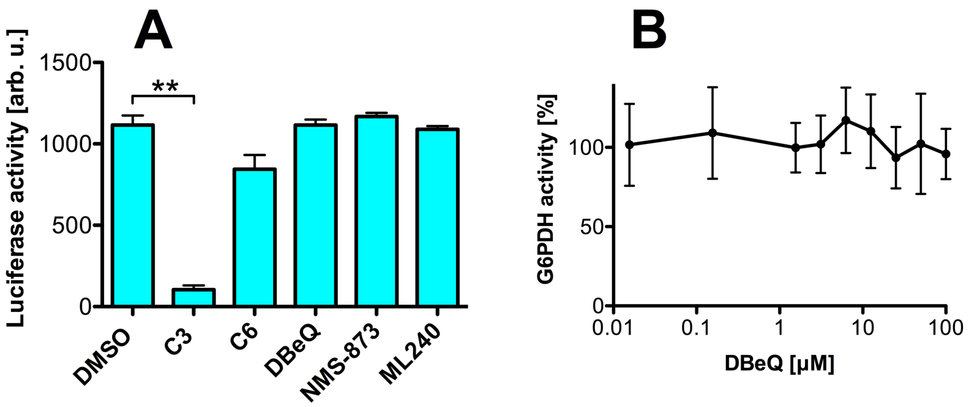

Supplementary Figure 7. Effects of the inhibitors on the activity of luciferase and glucose-6-phosphate dehydrogenase (G6PDH). (A) Firefly luciferase activity was determined in the absence (DMSO) or in the presence 100 µM ClpB inhibitor candidates. The only statistically significant difference is shown with ** ($p < 0.01$, $n = 2$). (B) G6PDH activity was determined in the presence of an increasing concentration of DBeQ and normalized to the DMSO control. Average values from two measurements are shown with standard deviations.

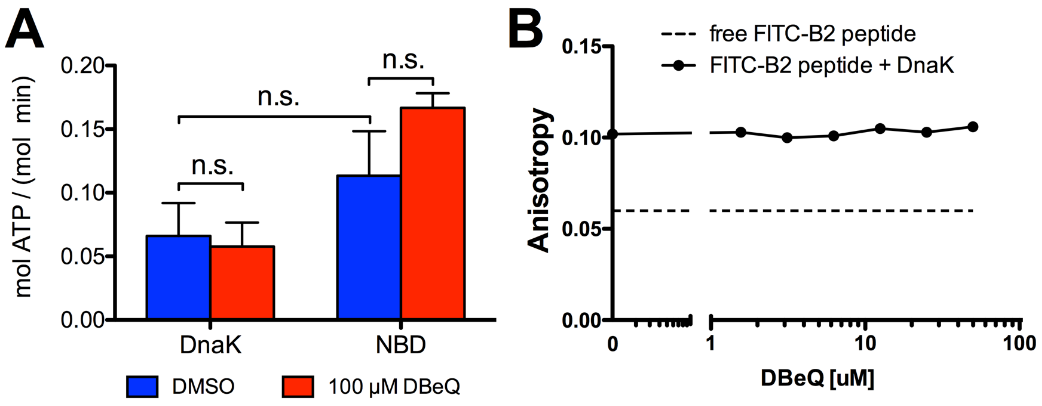

Supplementary Figure 8. Effects of DBeQ on DnaK. (A) The ATPase activity of DnaK and its nucleotide binding domain (NBD) in the absence of DBeQ (DMSO control, blue) and with 100 μM DBeQ (red). Shown are the values from 3 independent measurements with standard deviations (n.s. indicates that the difference between the data sets is not statistically significant, $p > 0.05$). (B) Fluorescence anisotropy of FITC-B2 (20 nM) in the absence (broken line) and presence of DnaK (solid line) at increasing DBeQ concentrations.

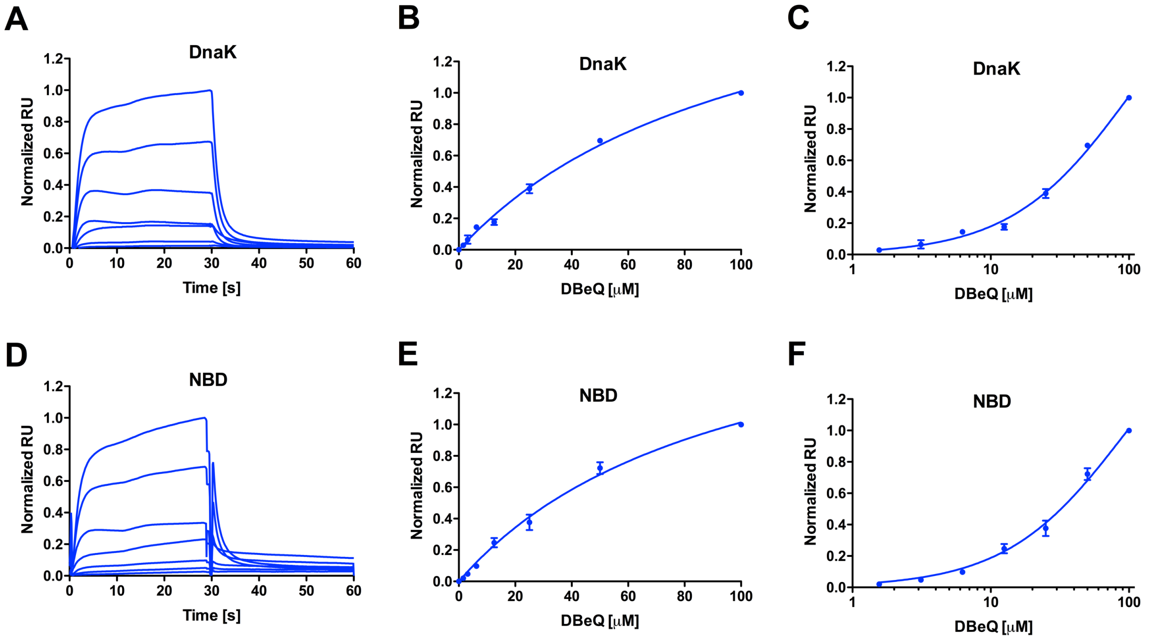

Supplementary Figure 9. **Surface Plasmon Resonance (SPR) analysis of the binding of DBeQ to DnaK (A-C) and its nucleotide binding domain (NBD) (D-F).** (A, D) Representative SPR sensograms for the DnaK variants. Each solid line represents a sensogram obtained with a given DBeQ concentration; a higher DBeQ concentration produces a higher SPR response. DBeQ binding isotherms are shown with the linear ligand concentration scale (B, E) or the logarithmic scale (C, F). Shown are the averages with standard deviations from three repeated experiments. Solid lines in the panels B, C, E, and F represent the fits of the non-cooperative binding model with the parameters listed in Supplementary Table 1.

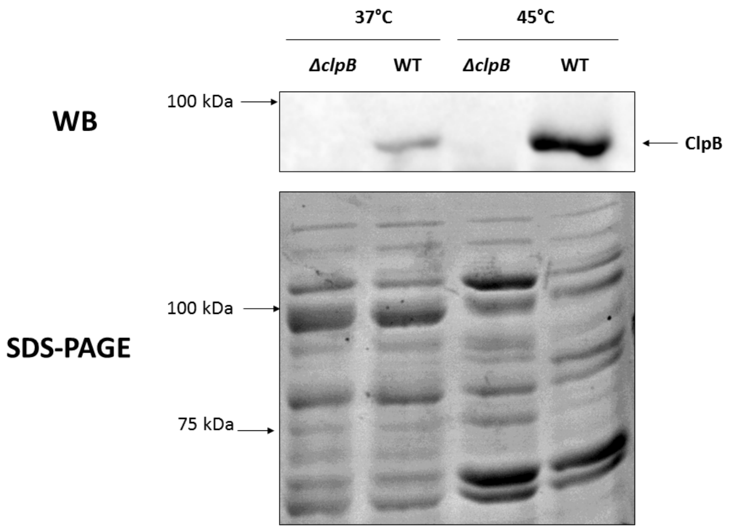

Supplementary Figure 10. Analysis of the ClpB protein levels in *E. coli.* Total cell lysates from MC4100 (WT) and MC4100*ΔclpB* (*ΔclpB*) *E. coli* strains grown at 37 °C and 45 °C were resolved with SDS-PAGE in an 8% gel. (Top) ClpB was detected using Western blotting. (Bottom) Coomassie Brilliant Blue staining of the same samples as in the top resolved in the control part of the same gel.

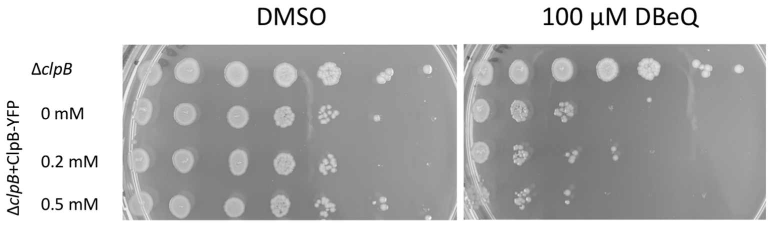

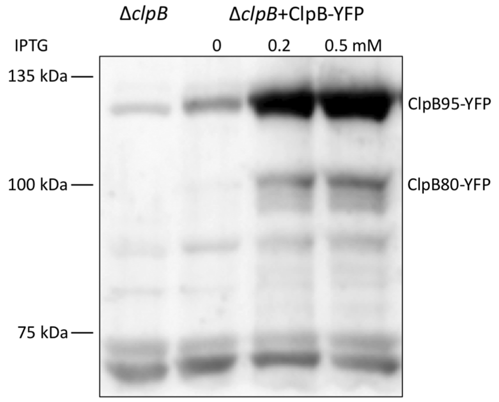

Supplementary Figure 11. Viability of *ΔclpB E. coli* strain upon expression of ClpB-YFP. (Top) The *E. coli* cultures were incubated at 45 °C in the presence of DBeQ or with only DMSO for 3 h then spotted on agar plates and incubated overnight at 37 °C. Expression of ClpB-YFP was induced with the IPTG concentration indicated on the left. Each spot on the agar plates represents a viable culture after a 10-fold serial dilution (from left to right). (Bottom) Western blot analysis of the bacterial lysates with anti-ClpB antibody. The cultures were incubated with the indicated IPTG concentration for 3 h. The detected signals correspond to the full-length ClpB-YFP (ClpB95-YFP) and the N-terminally truncated ClpB80-YFP (1,2). The non-specific band below 75 kDa serves as a loading control.

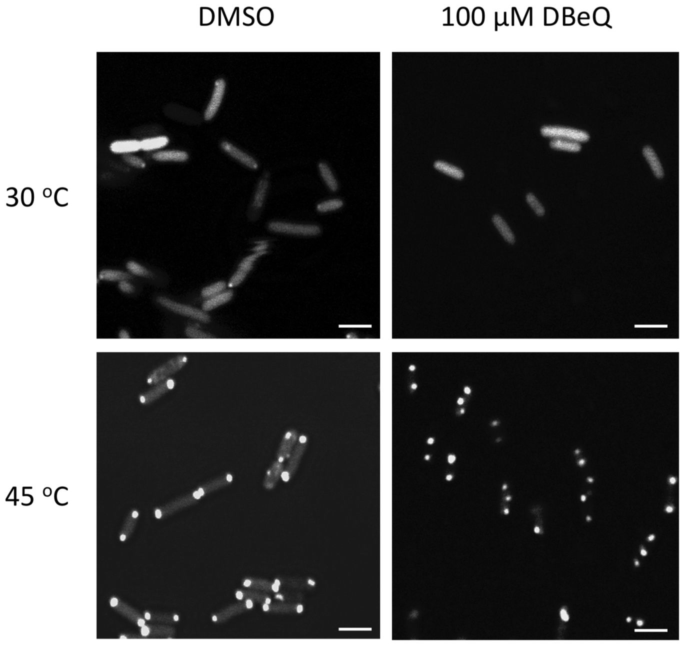

Supplementary Figure 12. Localization of YFP-luciferase in *E. coli* cells under heat shock. MC4100 cells were grown at 30 °C and then shifted to 45 °C for 30 min. The images show YFP fluorescence signal. Representative images from 3 independent experiments are shown. The white bar in each panel corresponds to 2 µm.

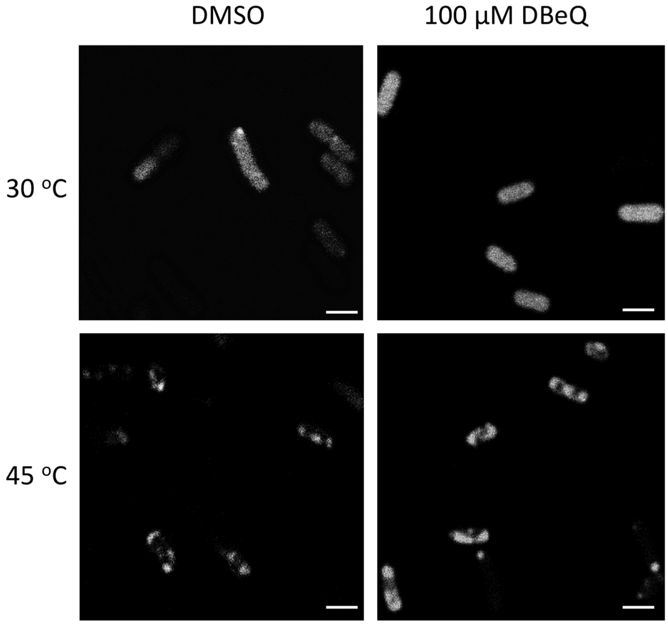

Supplementary Figure 13. Localization of ClpB-YFP in the *dnaK*756 *E. coli* strain under heat shock. MC4100 cells were grown at 30 °C and then shifted to 45 °C for 30 min. The images show YFP fluorescence signal. Representative images from 3 independent experiments are shown. The white bar in each panel corresponds to 2 µm.

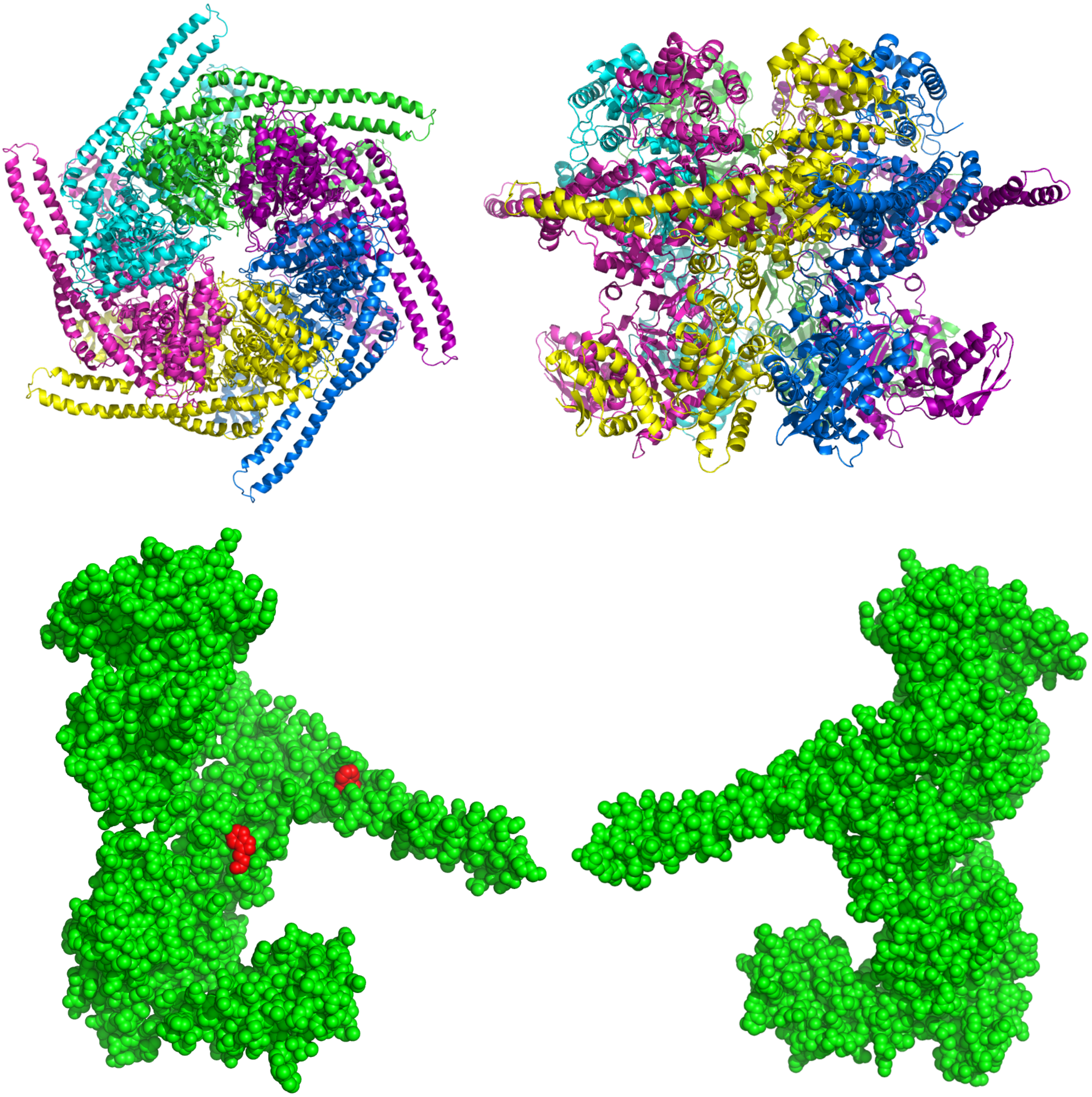

Supplementary Figure 14. (Top) Model of the ClpB hexamer (top and side view) assembled from the *Thermus thermophilus* ClpB monomer structure (PDB Id: 1QVR, chain A). Each subunit in the hexamer is shown in a different color. (Bottom) Space-filling model of the *T. thermophilus* ClpB monomer viewed from the inside of the hexamer (left) and from the outside of the hexamer (right). The side chains of the two Trp residues are shown in red (see Supplementary Figure 4). The indicated Trp residues in ClpB are conserved between *E. coli* and *T. thermophilus*.

References

1. Squires, C.L., Pedersen, S., Ross, B.M., and Squires, C. (1991) ClpB is the Escherichia coli heat shock protein F84.1. *J. Bacteriol.* **173**, 4254-4262
2. Chow, I.T., Barnett, M.E., Zolkiewski, M., and Baneyx, F. (2005) The N-terminal domain of Escherichia coli ClpB enhances chaperone function. *FEBS Lett.* **579**, 4242-4248
